# Association of EDARV370A with breast density and metabolic syndrome in Latinos

**DOI:** 10.1371/journal.pone.0258212

**Published:** 2021-10-07

**Authors:** Dawn K. Coletta, Leslea J. Hlusko, G. Richard Scott, Luis A. Garcia, Celine M. Vachon, Aaron D. Norman, Janet L. Funk, Gabriel Q. Shaibi, Valentina Hernandez, Eleanna De Filippis, Lawrence J. Mandarino

**Affiliations:** 1 Department of Medicine, Division of Endocrinology, University of Arizona, Tucson, Arizona, United States of America; 2 Department of Physiology, University of Arizona, Tucson, Arizona, United States of America; 3 Center for Disparities in Diabetes Obesity, and Metabolism, University of Arizona, Tucson, Arizona, United States of America; 4 Department of Integrative Biology, University of California, Berkeley, California, United States of America; 5 Centro Nacional de Investigación sobre la Evolución Humana, Burgos, Spain; 6 Department of Anthropology, University of Nevada, Reno, Nevada, United States of America; 7 Division of Epidemiology, Mayo Clinic, Rochester, Minnesota, United States of America; 8 Department of Nutritional Sciences, University of Arizona, Tucson, Arizona, United States of America; 9 Center for Health Promotion and Disease Prevention, Arizona State University, Phoenix, Arizona, United States of America; 10 Mountain Park Health Center, Phoenix, Arizona, United States of America; 11 Department of Endocrinology, Metabolism and Diabetes, Mayo Clinic Arizona, Scottsdale, Arizona, United States of America; Banaras Hindu University Faculty of Science, INDIA

## Abstract

The ectodysplasin receptor (EDAR) is a tumor necrosis factor receptor (TNF) superfamily member. A substitution in an exon of EDAR at position 370 (EDARV370A) creates a gain of function mutant present at high frequencies in Asian and Indigenous American populations but absent in others. Its frequency is intermediate in populations of Mexican ancestry. EDAR regulates the development of ectodermal tissues, including mammary ducts. Obesity and type 2 diabetes mellitus are prevalent in people with Indigenous and Latino ancestry. Latino patients also have altered prevalence and presentation of breast cancer. It is unknown whether EDARV370A might connect these phenomena. The goals of this study were to determine 1) whether EDARV370A is associated with metabolic phenotypes and 2) if there is altered breast anatomy in women carrying EDARV370A. Participants were from two Latino cohorts, the Arizona Insulin Resistance (AIR) registry and *Sangre por Salud* (SPS) biobank. The frequency of EDARV370A was 47% in the Latino cohorts. In the AIR registry, carriers of EDARV370A (GG homozygous) had significantly (p < 0.05) higher plasma triglycerides, VLDL, ALT, 2-hour post-challenge glucose, and a higher prevalence of prediabetes/diabetes. In a subset of the AIR registry, serum levels of ectodysplasin A2 (EDA-A2) also were associated with HbA1c and prediabetes (p < 0.05). For the SPS biobank, participants that were carriers of EDARV370A had lower breast density and higher HbA1c (both p < 0.05). The significant associations with measures of glycemia remained when the cohorts were combined. We conclude that EDARV370A is associated with characteristics of the metabolic syndrome and breast density in Latinos.

## Introduction

Ectodysplasin is a member of the tumor necrosis family (TNF) superfamily, and its receptor, the ectodysplasin receptor (EDAR), is a member of the TNF family of receptors [1]. Ectodysplasin signaling is involved in the embryonic development of many ectodermal tissues, including hair, sweat glands, mammary glands, and teeth [2]. Loss of function mutants of components of this system produces the syndrome of hypohydrotic ectodermal dysplasia [3]. A gain of function coding variant of EDAR [4], a single base substitution in an exon of the ectodysplasin receptor resulting in an alanine for a valine residue (EDARV370A), is present at high frequencies in North and East Asian populations and Indigenous populations of the Americas [5]. Individuals carrying at least one EDARV370A allele have a higher probability of having straight hair, more eccrine sweat glands, and shoveling of upper incisors [6–14]. A knock-in mouse with the EDARV370A allele mimics many of these characteristics (except incisor shoveling), along with an increased branch density of the mammary glands [6]. Genomic analysis indicates that EDAR and the surrounding region have undergone a high degree of natural selection in favor of EDARV370A over the last approximately 30,000 years [4, 15]. One speculation regarding this selection is that a higher number of sweat glands may have been adaptive in Asian hunter-gatherers [6].

Upper incisor shoveling is prevalent in Indigenous American and some Asian populations [16]. The causative relationship between EDARV370A and dental crown morphology has explained this phenomenon [11–13]. Hlusko suggests this allele conferred selective advantage during population movements across Beringia, involving potential genetic isolation, especially during periods when people may have had restricted access to adjacent continents due to glacial conditions [5]. In addition to speculation regarding a selective advantage related to sweat glands, another hypothesis is that because Beringia received very little UV-B radiation for much of the year, vitamin D synthesis in the skin of infants consuming only breastmilk would not have occurred at levels high enough to promote normal development of the infant. In this case, it may have been selectively advantageous if mothers could secrete more vitamin D (and other fat-soluble components) in their milk to supply their infants with adequate nutrition, and this may have provided a selective advantage to infants of mothers carrying the EDARV370A variant, perhaps due to higher mammary duct branch complexity in these women [5]. If the EDARV370A variant was already at a high frequency when East Asian populations migrated into ancient Beringia, it could have facilitated the success of Beringian people in that high latitude environment. However, other than the observation that there is an increased branch density of the mammary glands in EDARV370A knock-in mice [6], there has been no study to provide evidence that would support this notion in humans. Suppose this was the case, and women bearing this allele were to have different mammary gland anatomy and function. In this case, it could have a bearing on the health of infants in ancient Beringia and may be involved in the health of infants and mothers even in today’s environments.

Breast cancer is a leading cause of cancer death in Latinas [17]. Most [18, 19], but not all [20, 21] studies show that Latina women have a greater risk of breast cancer-specific mortality than non-Hispanic white women. However, age-adjusted breast cancer incidence rates are about 25% lower among Latina women than non-Hispanic white women [22]. These observations suggest that there may be biological differences, perhaps involving genetic differences, in breast cancer pathogenesis that distinguishes Latinas from non-Hispanic whites. The EDARV370A variant conceivably could be involved in the differences in presentation of breast cancer in Latinas. Since the mammographic determination of breast density is an independent risk factor for breast cancer, one goal of the present study was to determine if there is evidence of altered breast anatomy in Latina women carrying the EDARV370A variant.

Many Indigenous populations of the Americas have high frequencies of the EDARV370A variant and many metabolic syndrome characteristics related to obesity-associated insulin resistance and type 2 diabetes mellitus [23]. It is unclear whether the EDARV370A variant is associated with metabolic events or why this should be the case. Still, the high prevalence of obesity and diabetes coexisting with the high frequency of EDARV370A is intriguing. Therefore, the second purpose of this study was to determine whether EDARV370A is associated with metabolic syndrome phenotypes. Moreover, we assayed serum levels of ectodysplasin A2 to determine whether other elements of signaling through the EDARs were related to components of the metabolic syndrome.

To answer these questions, we genotyped the EDAR variant in two cohorts of Latino participants. One cohort consisted of Latinos recruited through the community who were participants of the Arizona Insulin Resistance registry [24], and the other consisted of Latino patients at Mountain Park Community Health Center who were participants of the *Sangre por Salud* (SPS) biobank [25]. The advantage of using Latino participants is that this population has a significant genetic contribution from both Indigenous and European alleles, so that there may be wide variation in the genotypes and phenotypes under study, which facilitates the association analysis. In addition, almost half of the participants from the SPS biobank had breast density measurements that we used as a proxy phenotype for differences in breast anatomy.

## Materials and methods

### Participants and cohorts

The Institutional Review Boards at Arizona State University, Mayo Clinic, and the University of Arizona approved the study. Participants took part in either of two cohorts. The first cohort, known as the Arizona Insulin Resistance (AIR) registry, consisted of Latino volunteers (n = 667). They were recruited directly through the community [24]. The majority of the 667 volunteers were adults 18 years or older (79.6%); however, children and adolescents (20.4%) participated in the study. Participants received a history and physical examination, laboratory determinations, and a 75g two-hour oral glucose tolerance test. Of these 667 participants, 94% consented to bank their DNA/serum and plasma for future studies without additional recontact. For the genotyping analysis for this present study, only the adult participants were studied (n = 502). The second cohort consisted of Latino patients (n = 997) in the *Sangre por Salud* (SPS) biobank [25]. SPS participants received laboratory determinations, a two-hour glucose tolerance test, and banked plasma/serum and DNA with consent to use de-identified data and biospecimens for future studies [25]. Additionally, 539 women who had participated in the SPS biobank also took part in the LLEAD (Latinas Learning about Breast Density) study [26], where participants received mammograms as part of the protocol. The Breast Imaging, Reporting and Data System (BI-RADS) scores and breast density values were obtained from the mammograms and used for the association analyses.

The IRB at Arizona State University approved the AIR registry study. Similarly, the IRB at Mayo Clinic approved the SPS biobank. Written consent was obtained on all AIR registry and SPS biobank participants. For the minors that were recruited into the AIR registry [although they were not studied as part of this project], written consent was obtained from their parents or guardians. Written consent was obtained for the AIR registry and SPS biobank for the banking of serum, DNA, and RNA to use deidentified data and biospecimens for future studies, like the one described herein. The University of Arizona approved the present study under protocol #1703255156. The present study was considered exempt by the ethics committee at the University of Arizona since it utilized deidentified information of previously consented banked samples, and no recontact was made with these participants.

### Single nucleotide polymorphism (SNP) genotyping

Genotyping for the EDARV370A (rs3827760) SNP was performed by the Assay-by-Design service (Applied Biosystems, Calif., USA), as previously described [27]. Briefly, in a 384-well plate, 2 μl of purified genomic DNA (2 ng/μl) were incubated with primers and probes with the SNP of interest (0.09 μl), 3.5 μl of TaqMan Universal Polymerase Chain Reaction Master Mix-No AmpErase UNG and 1.14 μl of distilled water. Samples were polymerase-chain-reaction-amplified on the Applied Biosystems 9700HT Thermal Cycler under the following conditions: denatured for 10 min at 95°C, denatured, annealed, and extended for 40 cycles of 15 s at 92°C and 1 min at 60°C. We scanned the 384-well microplates for fluorescence emission using a 7900HT sequence detector (Applied Biosystems) and determined the alleles using the allelic discrimination Sequence Detection System v2.3 software (Applied Biosystems). Our success rate for genotyping in the AIR registry was 100%, and for the SPS biobank, we were able to call 993 genotypes out of the 997 DNA samples (99.6%).

### AIR registry phenotypes used for genetic analysis

The following phenotypes used for the AIR registry EDARV370A association analysis included body mass index (BMI), fat mass percentage (determined by bioimpedance; fatmass), waist circumference (WC), hip circumference (HC), total cholesterol (Chol), triglyceride (TG), high-density lipoprotein-cholesterol (HDL), low-density lipoprotein-cholesterol (LDL), very low-density lipoprotein-cholesterol (VLDL), systolic blood pressure (SBP), diastolic blood pressure (DBP), alanine aminotransferase (ALT), aspartate aminotransferase (AST), adiponectin, fasting plasma glucose (FPG), 2-hour oral glucose tolerance test (2hOGTT), hemoglobin A1c (HbA1c), fasting plasma insulin (FPI), prediabetes status and diabetes status using American Diabetes Association criteria. Shaibi et al. [24] and DeMenna et al. [27] describe the collection and measurement of these phenotypes. As part of the present study, ectodysplasin A2 (EDA-A2) levels were measured in a subset of stored serum samples using ELISAs and per manufacturer’s instructions (RayBiotech Life, GA, USA).

### SPS biobank phenotypes for genetic analysis

The following phenotypes used for the SPS biobank EDARV370A association analysis included body mass index (BMI), waist circumference (WC), total cholesterol (Chol), triglyceride (TG), high-density lipoprotein (HDL), low-density lipoprotein (LDL), fasting plasma glucose (FPG), 2-hour oral glucose tolerance test (2hOGTT), hemoglobin A1c (HbA1c), fasting plasma insulin (FPI), breast imaging reporting and data system (BI-RADS) scores and breast density (BD) value. The BI-RADS is a numerical scale ranging between 0 and 6 and is related to the likelihood that mammographic findings represent a malignancy. The mammogram report also consists of the BD value, which is an assessment of breast density. Breast density categorization was as follows: 1, almost entirely fatty; 2, scattered areas of fibroglandular density; 3, heterogeneously dense, which may obscure small masses; and 4, extremely dense, which lowers the sensitivity of the mammography. In addition, we had additional phenotypic information for women with mammogram data, including the number of children delivered, age at menarche, menopause (yes/no), and did you ever breastfeed (yes/no).

### Statistical analysis

We used R version 4.0.5 (https://www.r-project.org/) for the statistical analysis. Mean ± SEM was used to express the participant characteristic data. The allele frequencies for EDARV370A (rs3827760) were calculated in R using the genetics package (https://CRAN.R-project.org/package=genetics). This same package was used to test Hardy-Weinberg Equilibrium (HWE). We used the linear model function in R to create a simple regression model for the genetic association phenotype analysis. Age, sex, and BMI were used as covariates in the EDAR variant phenotype association analyses. For the BI-RADS score and BD value association analyses, we included age, BMI, menopausal status, and the number of children delivered in the model. Significance was defined as p ≤ 0.05.

## Results

### AIR registry and SPS biobank participants

Table 1 shows the clinical characteristics of the AIR registry participants used in the present study. Phenotypic data were available on 502 participants (324 females, 178 males) with a mean age of 36.3 ± 0.5 years. The clinical characteristics of the SPS biobank participants also are provided in Table 1. Phenotypic data were available on 997 participants (691 females, 306 males) with a mean age of 45.8 ± 0.4 years. Moreover, 539 SPS women participants also had mammograms with breast density (BD) values and BI-RADS scores data.

**Table 1:** Characteristics of the AIR registry and SPS biobank participants.

	AIR registry	SPS biobank
**Gender (Female / Male)**	324 / 178	691 / 306
**Age, years**	36.3 ± 0.5	45.8 ± 0.4
**Body Mass Index, kg/m2**	29.8 ± 0.3	30.5 ± 0.2
**Fat Mass, %**	24.3 ± 0.5	–
**Waist circumference, cm**	98.6 ± 0.6	100.7 ± 0.5
**Hip circumference, cm**	108.6 ± 0.5	–
**Cholesterol, mg/dl**	174.3 ± 1.6	186.3 ± 1.2
**Triglycerides, mg/dl**	136.4 ± 3.5	144.8 ± 3.0
**High-density lipoprotein, mg/dl**	44.0 ± 0.5	49.9 ± 0.5
**Low-density lipoprotein, mg/dl**	106.9 ± 1.3	108.1 ± 1.0
**Very low-density lipoprotein, mg/dl**	21.8 ± 0.5	–
**Systolic blood pressure, mm Hg**	120.1 ± 0.7	–
**Diastolic blood pressure, mm Hg**	76.4 ± 0.4	–
**Alanine aminotransferase, IU/L**	26.5 ± 0.8	–
**Aspartate aminotransferase, IU/L**	24.1 ± 0.5	–
**Adiponectin, μg/ml**	6.4 ± 0.1	–
**Hemoglobin A1c, %**	5.59 ± 0.02	6.11 ± 0.04
**Fasting plasma insulin, μIU/ml**	9.1 ± 0.3	10.3 ± 0.6
**Fasting plasma glucose, mg/dl**	94.4 ± 0.5	95.2 ± 0.7
**2hOGTT, mg/dl**	136.8 ± 2.3	122.4 ± 1.9
**Diabetes status, %**	14.9 ± 1.6	–
**Prediabetes status, %**	36.4 ± 2.4	–

Values are mean ± SEM. The–indicates that this phenotype was unavailable for this dataset.

For additional detail, the S1 and S2 Tables shows the AIR registry and SPS biobank characteristic data organized by EDARV370A genotype and body mass index (BMI).

### Minor allele frequency and HWE calculation

EDARV370A (rs3827760) had an allele frequency of approximately 47% in each cohort. In the AIR registry cohort, genotype frequencies (AA: 141, AG: 254: GG: 107) met Hardy-Weinberg equilibrium criteria (Chi-squared test, p = not significant). However, genotype frequencies (AA: 299, AG: 446: GG: 248) in the SPS biobank were inconsistent with Hardy-Weinberg equilibrium (Chi-squared test, p = 0.0017). Similarly, when we combined the genotype frequencies (AA: 440, AG: 700: GG: 355) from both Latino cohorts, Hardy-Weinberg equilibrium was not met (Chi-squared test, p = 0.019). A departure from Hardy-Weinberg equilibrium for EDARV370A (rs3827760) was not unexpected since this variant may have undergone a high degree of natural selection within the last 30,000 years [5].

### AIR registry association analyses

Table 2 provides the mean levels for the phenotypes having significant associations with EDARV370A. EDARV370A in the AIR registry was significantly associated with TG, VLDL, ALT, 2hOGTT, prediabetes, and diabetes status (Table 2). Carriers of the alanine variant EDARV370A/ EDARV370A (GG homozygous) had higher TG, higher VLDL, higher ALT, higher 2-hour post-challenge glucose, and more prediabetes/diabetes compared with carriers of the EDARwt/EDARwt (AA homozygous).

**Table 2:** Genotype class specific mean values for the phenotypes that were significantly associated with EDARV370A.

Phenotype	EDARwt / EDARwt (AA)	EDARwt / EDARV370A (AG)	EDAR370A / EDARV370A (GG)	Direction of Change	Genotype only model P Value*	Genotype, age, sex and BMI model P Value**
Triglycerides (mg/dl)	120.0 ± 0.5	136.5 ± 0.3	157.8 ± 0.9	↑	**0.000167**	**0.00039**
VLDL (mg/dl)	20.1 ± 0.1	22.2 ± 0.1	23.4 ± 0.1	↑	**0.0173**	**0.018**
ALT (IU/L)	24.0 ± 0.1	26.2 ± 0.1	30.7 ± 0.2	↑	**0.00392**	**0.00807**
2-hour post-challenge glucose	128.1 ± 0.4	136.8 ± 0.2	148.6 ± 0.6	↑	**0.00214**	**0.001137**
Prediabetes (%)	29.2 ± 0.4	38.0 ± 0.2	42.7 ± 0.6	↑	**0.042**	0.058
Diabetes (%)	12.4 ± 0.2	13.3 ± 0.1	21.9 ± 0.4	↑	**0.0511**	**0.0269**

The p values were generated using the simple linear regression model in R.

*Genotype only was included in the linear regression model.

**Genotype, age, sex and BMI were included in the linear regression model. For ease of interpretation, the genotype class specific mean ± SEM are shown along with the direction of change that corresponds to the EDARV370A / EDARV370A (GG) genotype. Significant p values are bolded.

The significant association for ALT appears to be driven by the males in the analysis. ALT levels across the three genotypes showed that the males had a significant association (AA: 26.5 ± 1.7 *versus* AG: 32.3 ± 1.9 *versus* GG: 43.1 ± 3.8 IU/L, p < 0.05) whereas the females were unchanged (AA: 22.8 ± 1.7 *versus* AG: 23.0 ± 1.2 *versus* GG: 22.3 ± 1.7 IU/L, NS). Moreover, the AST levels in the males had a significant association with the EDAR genotype (AA: 23.4 ± 1.1 *versus* AG: 25.2 ± 1.1 *versus* GG: 31.2 ± 1.9 IU/L, p < 0.05), whereas the females did not (AA: 23.2 ± 1.2 *versus* AG: 23.1 ± 0.8 *versus* GG: 22.1 ± 1.1 IU/L, NS). Of note, the trend for all phenotypes was in the direction of the EDARV370A homozygous participants having more metabolic syndrome characteristics (Table 2).

### SPS biobank analysis

EDARV370A in the SPS biobank was significantly associated with HbA1c (p = 0.050). When we included age, sex, and BMI in the model, the P value fell to 0.0167. Participants that were EDARwt/EDARwt (AA homozygous), EDARwt/EDARV370A (AG heterozygous), and EDARV370A/ EDARV370A (GG homozygous) had HbA1c levels of 5.98 ± 0.06%, 6.16 ± 0.06%, and 6.20 ± 0.09%, respectively (higher HbA1c in EDARV370A/EDARV370A carriers (GG homozygous). EDARV370A was not associated with any of the other metabolic phenotypes in the SPS biobank.

We also performed analysis to determine the relationship between breast density (BD), as determined using mammography, with EDAR genotypes. There was a significant inverse association of the EDARV370A genotype with BD value (p = 0.0312). When we included age and BMI in the model, the significance improved (p = 0.0066). Participants that were EDARwt/EDARwt (AA homozygous), EDARwt/EDARV370A (AG heterozygous), and EDARV370A/EDARV370A (GG homozygous) had a BD value of 2.46 ± 0.05, 2.43 ± 0.04, and 2.30 ± 0.05, respectively. Correction for BMI was important in the model because of an independent inverse association between breast density and BMI (p < 0.000001). Lean, overweight, and obese participants had BD values of 2.69 ± 0.07, 2.50 ± 0.04, and 2.29 ± 0.03, respectively. Similarly, the correction for age was also important because of an independent association of breast density with age (p < 0.000001). The average age (±SEM) of the women across the four breast density categories was 51.7 ± 2.0, 51. 3 ± 0.5, 48.0 ± 0.5 and 43.4 ± 0.9 years for the 1 = almost entirely fatty, 2 = scattered areas of fibroglandular density, 3 = heterogeneously dense and 4 = extremely dense groups, respectively.

In addition, we showed that BD value was independently associated with menopausal status (p = 0.000618). The BD value was lower in the menopausal women (2.28 ± 0.04) than the non-menopausal women (2.48 ± 0.04). We also showed that the BD value was independently associated with the number of children delivered (p = 0.00248). Across the four BD categories of 1, 2, 3, and 4, the average number of children delivered (±SEM) was 3.4 ± 0.3, 3.7 ± 0.1, 3.3 ± 0.1, and 2.6 ± 0.5, respectively. Breast density was not associated with age at menarche (p = 0.263) nor breastfeeding history (p = 0.333). Based on these findings, we reran our analysis of the BD value with the EDAR genotypes while controlling for the significant independent traits (age, BMI, menopausal status, and the number of children delivered). After controlling for these other factors, the genotype association with BD value remained with p = 0.0218.

### AIR registry and SPS biobank combined analysis

Table 3 summarizes the p values derived for the EDARV370A association analysis by phenotypes for AIR registry, SPS biobank, and AIR + SPS combined.

**Table 3:** Summary table of the EDARV370A association analysis by phenotypes for AIR registry, SPS biobank and AIR + SPS combined.

	AIR registry P Value	SPS biobank P Value	AIR + SPS combined P Value
Gender (Female / Male)	NS	NS	NS
Age, years	NS	NS	NS
Body Mass Index, kg/m2	NS	NS	NS
Fat Mass, %	NS	–	–
Waist circumference, cm	NS	NS	NS
Hip circumference, cm	NS	–	–
Cholesterol, mg/dl	NS	NS	NS
Triglycerides, mg/dl	0.000167* / 0.00039**	NS	NS
High-density lipoprotein, mg/dl	NS	NS	NS
Low-density lipoprotein, mg/dl	NS	NS	NS
Very low-density lipoprotein, mg/dl	0.0173* / 0.018**	–	–
Systolic blood pressure, mm Hg	NS	–	–
Diastolic blood pressure, mm Hg	NS	–	–
Alanine aminotransferase, IU/L	0.00392* / 0.00807**	–	–
Aspartate aminotransferase, IU/L	NS	–	–
Adiponectin, μg/ml	NS	–	–
Hemoglobin A1c, %	NS	0.050* / 0.0167**	0.018* / 0.010**
Fasting plasma insulin, μIU/ml	NS	NS	NS
Fasting plasma glucose, mg/dl	NS	NS	0.062* / 0.036**
2-hour post-challenge glucose, mg/dl	0.00214* / 0.001137**	NS	NS
Diabetes status, %	0.0511*/ 0.0269**	–	–
Prediabetes status, %	0.042* **/** 0.058**	–	–
Breast density (BD) value	–	0.0312* / 0.0066*** / 0.0218****	–
BI-RADS Score	–	NS	–

The–indicates that this phenotype was unavailable for this dataset. NS = not significant. The p values were generated using the simple linear regression model in R.

*Genotype only was included in the linear regression model.

**Genotype, age, sex and BMI were included in the linear regression model.

***Genotype, age and BMI were included in the linear regression model.

****Genotype, age, BMI, menopausal status and number of children delivered were included in the linear regression model. Significant p values are bolded.

**Fig 1:**
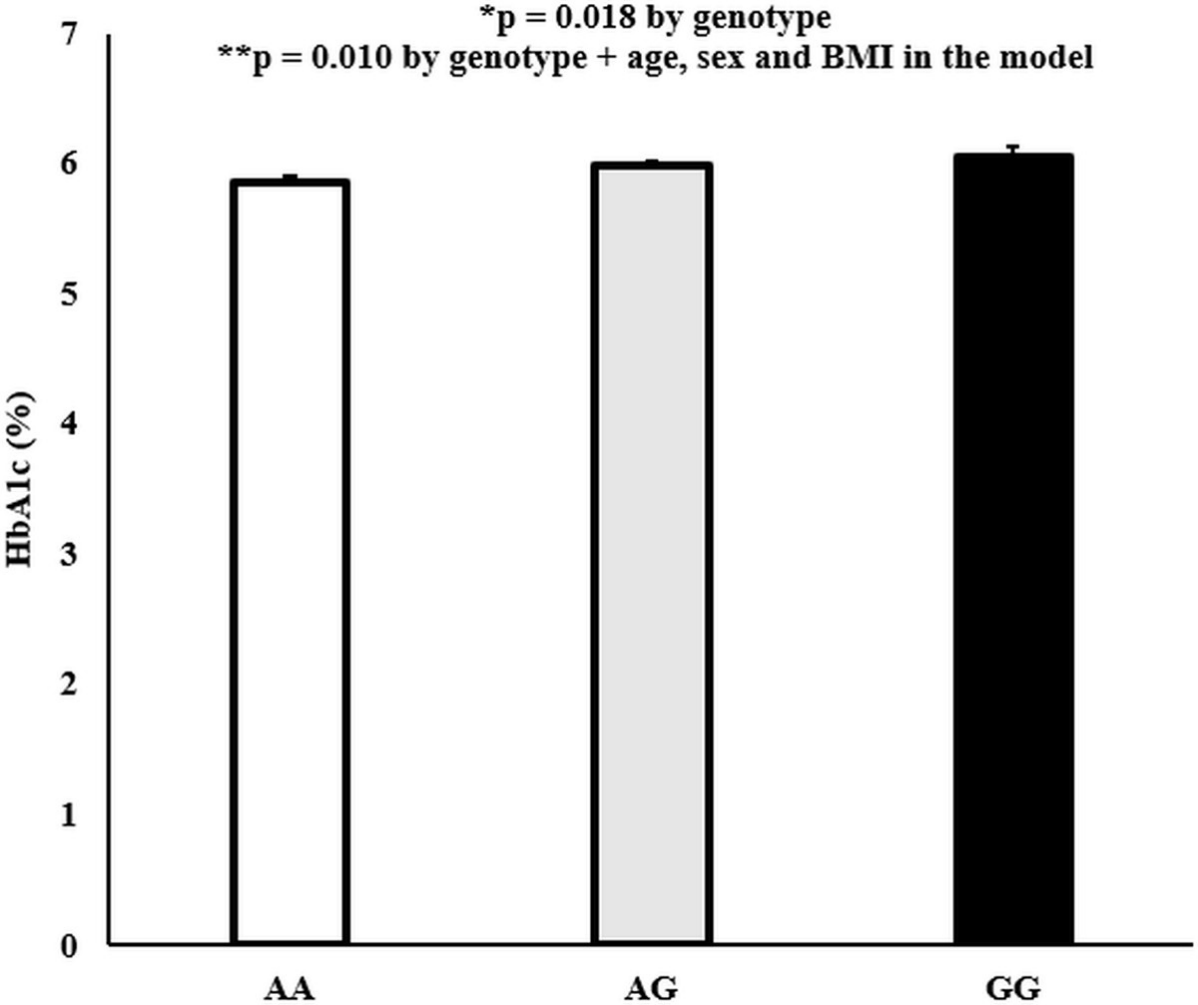
EDARV370A genotypes by HbA1c (%) levels. Values are mean ± SEM. P values were generated using a simple linear regression model.

**Fig 2:**
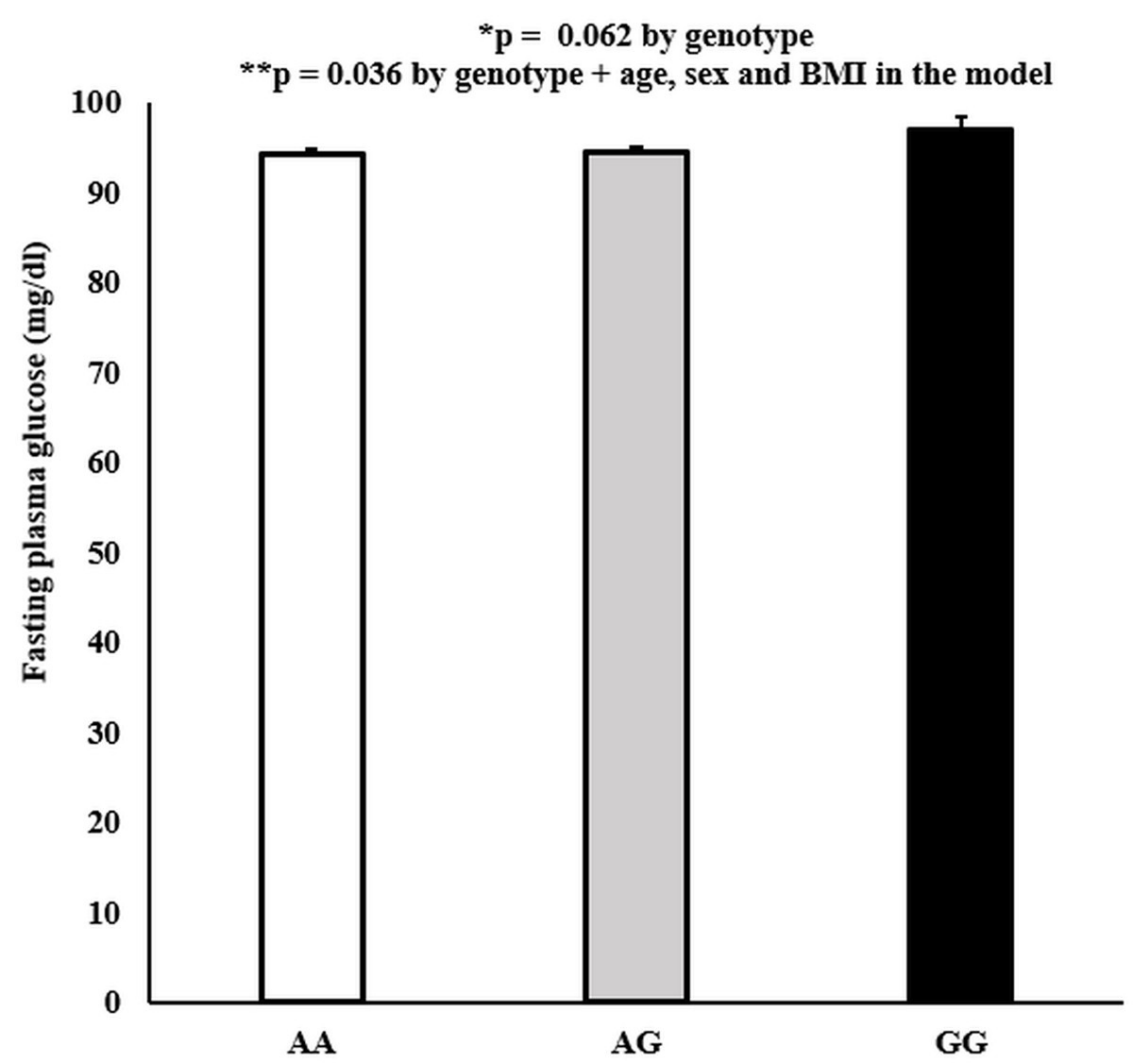
EDARV370A genotypes by fasting plasma glucose (mg/dl) levels. Values are mean ± SEM. P values were generated using a simple linear regression model.

We had phenotypic data on 1,499 participants aged 18 to 85 years (1,015 females and 484 males) for the combined AIR registry and SPS biobank analysis. The average age was 42.6 ± 0.3 years. EDARV370A was significantly associated with measures of glycemia (higher HbA1c and FPG in alanine carriers of EDARV370A/EDARV370A (GG homozygous)). In addition, there was a significant association of the EDARV370A genotype with HbA1c (p = 0.018). After controlling for age, sex, and BMI in the model, the significance was p = 0.010 (Fig 1). Moreover, there was a nominal association of the EDARV370A genotype with FPG (p = 0.062), and after including age, sex, and BMI in the model, the significance was p = 0.036 (Fig 2). Additionally, the associations for HbA1c and FPG were driven by the females in the analysis. When we analyzed the data by gender and genotypes, we showed significant associations in the females but not the males (S3 Table).

### Ectodysplasin A2 (EDA-A2) analysis

**Fig 3:**
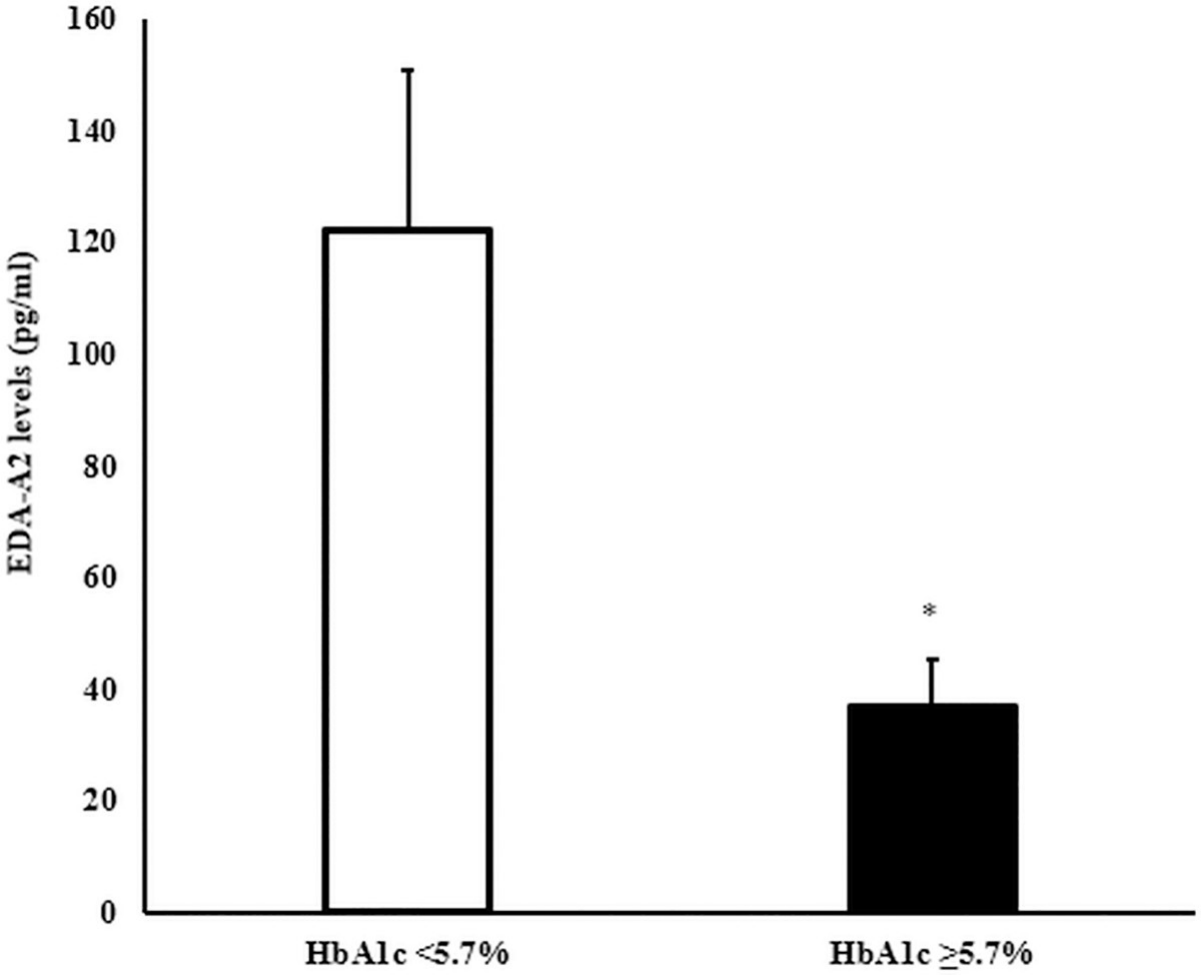
EDA-A2 levels (pg/ml) by HbA1c cutoffs of normal glucose tolerance <5.7% and prediabetes ≥5.7%. Values are mean ± SEM. *p < 0.05.

**Fig 4:**
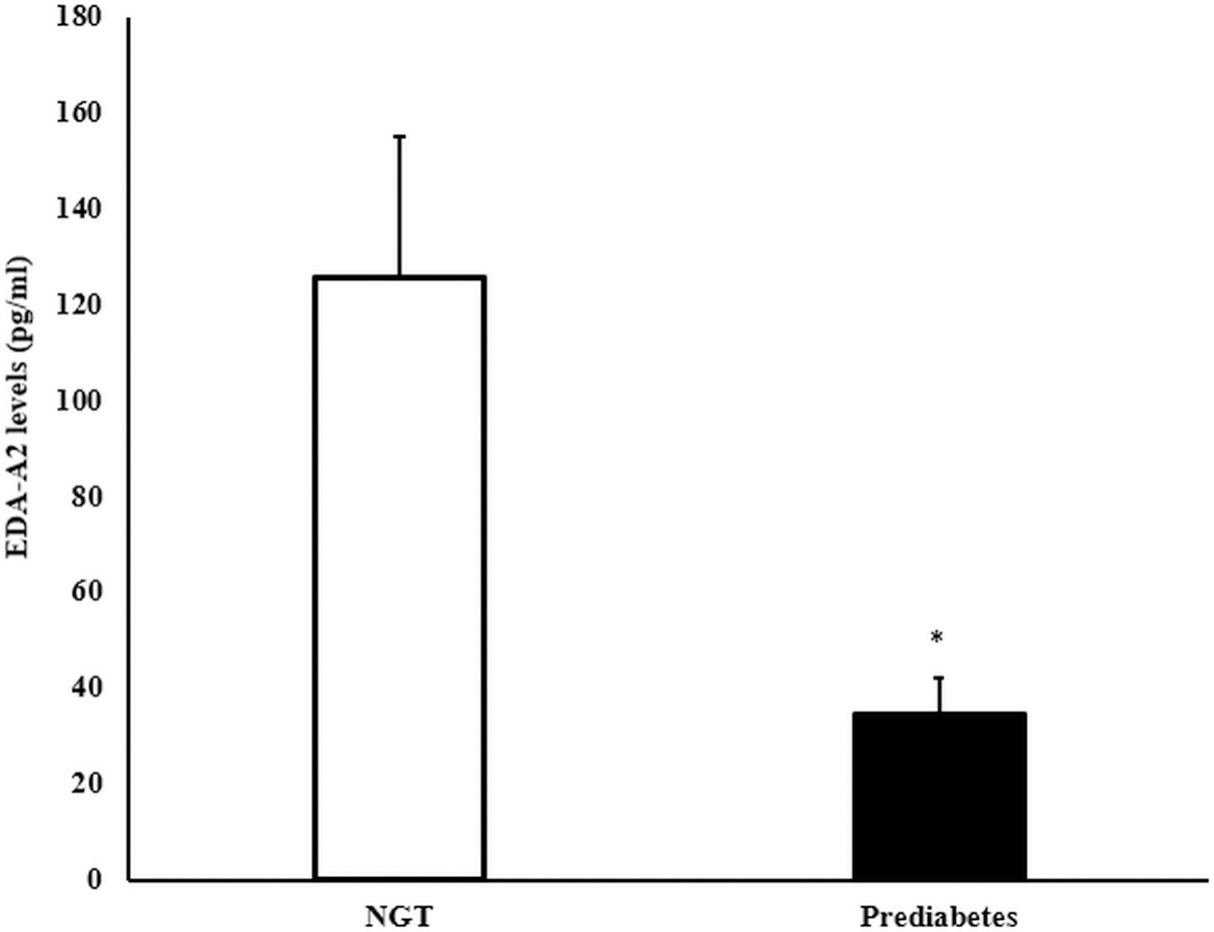
EDA-A2 levels (pg/ml) by prediabetes status. Values are mean ± SEM. *p < 0.05.

As part of the present study, we measured ectodysplasin A2 (EDA-A2) in a subset of sera from patients in the AIR registry (n = 234). We attempted to match for an equal number of sera in each genotype group, and we measured randomly across the adult patients’ samples. Mean serum EDA-A2 was 91.7 ± 17.5 pg/ml in this subset. We performed an association analysis of the EDA-A2 levels with the AIR registry metabolic phenotypes. There were significant associations of the serum EDA-A2 with HbA1c (Fig 3) and prediabetes (Fig 4). However, EDA-A2 levels were not associated with the EDAR genotype (S4 Table). This finding was not unexpected since this isoform of EDA binds to a related but distinct, X-linked ectodysplasin-A2 receptor (XEDAR) [28].

### Community advisory board (CAB) input

As part of the SPS biobank, a community advisory board (CAB) was constituted to seek community and patient input on biobank policies, procedures, and community implications of findings of studies using biobank resources [25]. Because the current project deals with potentially sensitive issues of evolution, that is, people of the Americas and Indigenous ancestry in Latinos, we sought to determine whether members of the CAB perceived areas of concern. To accomplish this, we presented and summarized the project and findings of this present study to the CAB. Following the presentation, members of the CAB, who are part of the Latino community, were asked to feel the “back of their upper front teeth” to detect incisor shoveling and, based on what they felt, to discuss their thoughts and feelings regarding how ancient ancestry might affect their health today and whether there were benefits or risks of such a study that might be of particular concern for the Latino community. One CAB member described an understanding of the “thrifty genotype” hypothesis [29], which they considered similar to this project. This CAB member considered genes derived from ancient ancestors as important, particularly as they may influence his current or future health status. Another CAB member said that although he identified as a “Chicano”, he understood that there is prejudice in the Latino community against “Indios” and related ancestry among some people, especially Mexicans (as differentiated from Chicano). The notion was that some members of his community might not appreciate the association to Indigenous ancestry. However, he felt that most “Chicanos” understand they are members of a distinct community, blended culturally and genetically, and probably would derive no offense from this study. “More information is almost always better” was the idea that was generally in agreement among CAB members.

## Discussion

The purposes of the present study were to determine whether the EDARV370A variant present in Latinos is associated 1) with phenotypic characteristics suggesting a connection between the variant and obesity, hyperglycemia, and other traits associated with the metabolic syndrome and 2) with alterations in breast characteristics as determined by mammography. EDARV370A was associated with various metabolic syndrome measures in two independent Latino cohorts and was significantly and inversely associated with breast density in women from the SPS biobank. The allele frequency of the EDARV370A allele in the present study was 47%, combining both cohorts. The EDARV370A variant allele is present at high frequencies in many Indigenous populations but is nearly absent in African or European people [5]. Therefore, we expected an intermediate allele frequency in the Latinos since they have major genetic contributions from Indigenous and European alleles. Overall, conditions for Hardy-Weinberg equilibrium at this locus were not met. Departure from Hardy-Weinberg equilibrium can result from genetic drift, population admixture, and natural selection. The population studied here is a result of recent admixture between Europeans and Indigenous Americans, and natural selection may have operated at this locus when ancestral populations resided in Beringia [5]. Moreover, genetic drift may have occurred in ancestral populations that may have also been genetically isolated [5]. Departures from Hardy-Weinberg equilibrium also are used to screen for genotyping errors [30], and although this is possible, it is unlikely since we used a candidate SNP genotyping approach. Moreover, the majority of genotyping errors are typically observed as an excess of heterozygotes, reflecting a technical problem in the assay [31]. This was not the case here.

One significant finding of this study is that the EDARV370A variant is significantly associated with breast density in Latinas. In this analysis, women who were homozygous for EDARV370A had lower breast density values, which implies less dense breast tissue and a greater proportion of adipose tissue. This finding was independent of adiposity. Developmentally, the knock-in mouse model of the EDARV370A variant had increased ductal branching in combination with a smaller fat pad area, as determined by whole-mount preparations of mammary glands [6]. This knock in mouse finding led to the hypothesis that EDARV370A’s effect on the mammary gland may have conferred selective advantage at high latitudes during population migrations to the Americas, 25–30,000 years BP, by conferring greater fitness to mothers who could transfer more macro and micronutrients and vitamins, such as vitamin D, to their infants [5]. From the mouse model, we might have predicted lower breast density in women carrying EDARV370A. However, mammograms provide only a rough picture of breast anatomy and do not have the resolution needed to image mammary ducts in women, so this study could not directly answer whether EDARV370A conferred an altered mammary gland branching in women who carry the allele. In contrast, mammary glands in the mouse could be examined histologically. Therefore, our finding of the association between EDARV370A and lower breast density was unexpected. However, the present results provide the first evidence that women carrying this allele, especially those who are homozygous, do have altered breast anatomy. In this case, a lower breast density value suggests a greater content of fatty tissue. Taken together, our association between this variant and differences in breast anatomy is intriguing. We suggest that this phenomenon needs additional study, as it could have a bearing on infant nutrition and even breast cancer. Evidence shows that very low breast density may be associated with worse outcomes in breast cancer patients [32]. It is important to note that this analysis corrected for BMI in these women. We found that BMI and breast density were independently and negatively associated; that is, more obese women had more fatty-rich breasts, consistent with prior population-based studies, such as the US Breast Cancer Surveillance Consortium [33].

If the hypothesis that greater mammary gland duct complexity can lead to higher breast milk content of fatty acids and fat-soluble vitamins, such as vitamin D [5], it also is conceivable that a genetic propensity in favor of higher overall body adiposity could interact with this trait to confer an additional selective advantage. For example, vitamin D is stored in adipose tissue, and the greater adipose tissue mass in obesity results in greater whole-body vitamin D stores, even though serum levels of vitamin D may be lower [34, 35]. Moreover, supplementation with vitamin D raises both plasma and adipose tissue vitamin D concentrations [36, 37], and this response is durable even after cessation of supplementation [38, 39]. Moreover, mammary gland adipocytes can form bioactive 1.25 hydroxyvitamin D from 25-hydroxyvitamin D [40]. In addition, Vitamin D receptor deletion results in longer mammary ducts (whereas the EDARV370A in mice increased branch density of the mammary glands), so the EDAR variant could counter the effects of vitamin D deficiency in mothers as well [41]. Such could also be the case for other metabolites, vitamins, or hormones stored in fat and secreted in breast milk.

Another major finding of our study is the evidence that carriers of the EDARV30A variant have several characteristics that are associated with the metabolic syndrome, including higher triglycerides, higher VLDL cholesterol, higher 2-hour post-challenge glucose, and higher percentages of prediabetes and diabetes, which both were present at nearly two-fold higher levels in Latinos homozygous for the EDARV370A allele. We also observed associations of this variant with measures of liver function, however that appeared to be male-specific. Thus, the EDARV370A variant is associated not only with differences in breast anatomy but also in metabolic traits. It is unclear whether there is an unknown mechanistic connection between the EDAR variant and metabolism or whether it serves as a marker for Indigenous ancestry, with the metabolic phenotypes being the product of other alleles more common in Indigenous populations.

To more completely examine the EDAR signaling system, we assayed an isoform of the ligand EDA in plasma of the participants. EDA-A1 and EDA-A2 are two isoforms of EDA that differ by an insertion of two amino acids [28]. This insertion determines the receptor that the ligand binds to. Specifically, EDA-A1 binds to the receptor EDAR, whereas EDA-A2 binds to the related X-linked ectodysplasin-A2 receptor (XEDAR) [28]. Although EDA-A2 is not a known ligand for EDAR, EDA-A2 levels were also measured in light of the finding of Yang et al. that levels of this ligand are associated with metabolic syndrome traits [42]. Specifically, they showed that EDA-A2 levels were lower in control participants compared to NAFLD patients [42]. In this study, we observed lower serum EDA-A2 levels with prediabetes and HbA1c. This finding was opposite to what we expected as the Yang et al. paper showed that EDA-A2 levels were higher in the NAFLD, which is associated with metabolic syndrome traits [42]. In this study, we did not observe any association of EDA-A2 with measures of liver function, specifically ALT or AST. This discrepancy may be explained by the cohorts studied. In the Yang et al. paper, the patients were from China [42], and our study focused on Latino volunteers. Unsurprisingly, there was no association of the EDA-A2 with the EDARV370A genotypes.

In addition to the evolutionary implications of the high frequency of the EDARV370A allele in Western Hemisphere populations, the results of this study may have health implications to carriers of this variant, including a higher risk of prediabetes/type 2 diabetes mellitus, potentially altered composition of breast milk that could have unknown health effects on infants, and even the speculative possibility that altered anatomy of mammary ducts could have an impact on the development of breast cancer. The frequency of EDARV370A is extremely high in Indigenous populations. In addition, with a calculated allele frequency of 0.47, we can speculate that about 70% of Mexican Americans, or 25–28 million individuals, also carry at least one EDARV370A allele. The potential magnitude of these unknown health effects compels us to gain a complete understanding of the biology of this gain of function variant.

Finally, genetic analysis in underserved minority communities has created sensitivities through misunderstanding and miscommunication, for example, in the case of genetic studies of the Havasupai [43]. In consideration of these sensitivities, the SPS biobank’s community advisory board (CAB) was created to provide input to investigators regarding potential sensitivities of Latino participants and the community regarding genetic research using DNA stored in the biobank. The CAB was engaged in assessing the type and extent of any such sensitivities to the conduct and findings of the present study. Because Latinos have major genetic contributions from Indigenous American and European populations and are distinct culturally from both, we deemed it essential to address these questions directly in representatives of the community. The study was described to the CAB and a discussion focused on how genetic findings related to ancient ancestry might affect health was undertaken. Generally, the members agreed that they would rather have this knowledge, regardless of its implications regarding ancestry, than not have the information that could affect their health. However, there was a discussion about how some Latino community members may have mixed feelings about Indigenous ancestry and that some participants might not want to have those kinds of results returned to them.

The findings of our study should be considered in the context of the strengths and limitations of this work. A main limitation of this study is that we did not measure ancestry informative markers (AIMs) in the AIR registry or the SPS biobank. As such, we were unable to assess the proportion of ancestry of individuals in our populations. In addition, we were not able to account for bias that may stem from genetic admixture or population stratification. On the other hand, a strength of this study was the access to the AIR registry and SPS biobank, which comprised self-reported participants of Latino descent. A second strength was the participants’ rich phenotyping, including the breast density data from the SPS biobank. In conclusion, the results of this study provide evidence of links between the gain-of-function EDARV370A variant, breast adiposity, and metabolism. Whether there are mechanistic links, for example, with breast anatomy, or whether these associations only exist as markers of Indigenous ancestry will require additional study.

## Supporting information

S1 Table AIR registry characteristic data organized by EDARV370A genotype and body mass index (BMI). Values are mean ± SEM.
(PDF)

S2 Table SPS biobank characteristic data organized by EDARV370A genotype and body mass index (BMI). Values are mean ± SEM.
(PDF)

S3 Table Gender and genotype class specific mean values for the glycemic traits in the combined AIR registry + SPS biobank. (PDF)

S4 Table Genotype class specific mean values for EDA-A2 levels. Values are mean ± SEM.
(PDF)

## References

[1] Sadier A, Viriot L, Pantalacci S, Laudet V. The ectodysplasin pathway: from diseases to adaptations. Trends Genet. 2014;30(1):24–31. doi: 10.1016/j.tig.2013.08.006 24070496

[2] Chang SH, Jobling S, Brennan K, Headon DJ. Enhanced Edar signalling has pleiotropic effects on craniofacial and cutaneous glands. PLoS One. 2009;4(10):e7591. doi: 10.1371/journal.pone.0007591 19855838 PMC2762540

[3] Goodwin AF, Larson JR, Jones KB, Liberton DK, Landan M, Wang Z, et al. Craniofacial morphometric analysis of individuals with X-linked hypohidrotic ectodermal dysplasia. Mol Genet Genomic Med. 2014;2(5):422–9. doi: 10.1002/mgg3.84 25333067 PMC4190877

[4] Bryk J, Hardouin E, Pugach I, Hughes D, Strotmann R, Stoneking M, et al. Positive selection in East Asians for an EDAR allele that enhances NF-kappaB activation. PLoS One. 2008;3(5):e2209. doi: 10.1371/journal.pone.0002209 18493316 PMC2374902

[5] Hlusko LJ, Carlson JP, Chaplin G, Elias SA, Hoffecker JF, Huffman M, et al. Environmental selection during the last ice age on the mother-to-infant transmission of vitamin D and fatty acids through breast milk. Proc Natl Acad Sci U S A. 2018;115(19):E4426–E32. doi: 10.1073/pnas.1711788115 29686092 PMC5948952

[6] Kamberov YG, Wang S, Tan J, Gerbault P, Wark A, Tan L, et al. Modeling recent human evolution in mice by expression of a selected EDAR variant. Cell. 2013;152(4):691–702. doi: 10.1016/j.cell.2013.01.016 23415220 PMC3575602

[7] Kowalczyk-Quintas C, Schneider P. Ectodysplasin A (EDA)—EDA receptor signalling and its pharmacological modulation. Cytokine Growth Factor Rev. 2014;25(2):195–203. doi: 10.1016/j.cytogfr.2014.01.004 24508088

[8] Fujimoto A, Ohashi J, Nishida N, Miyagawa T, Morishita Y, Tsunoda T, et al. A replication study confirmed the EDAR gene to be a major contributor to population differentiation regarding head hair thickness in Asia. Hum Genet. 2008;124(2):179–85. doi: 10.1007/s00439-008-0537-1 18704500

[9] Tan J, Yang Y, Tang K, Sabeti PC, Jin L, Wang S. The adaptive variant EDARV370A is associated with straight hair in East Asians. Hum Genet. 2013;132(10):1187–91. doi: 10.1007/s00439-013-1324-1 23793515

[10] Peng Q, Li J, Tan J, Yang Y, Zhang M, Wu S, et al. EDARV370A associated facial characteristics in Uyghur population revealing further pleiotropic effects. Hum Genet. 2016;135(1):99–108. doi: 10.1007/s00439-015-1618-6 26603699

[11] Park JH, Yamaguchi T, Watanabe C, Kawaguchi A, Haneji K, Takeda M, et al. Effects of an Asian-specific nonsynonymous EDAR variant on multiple dental traits. J Hum Genet. 2012;57(8):508–14. doi: 10.1038/jhg.2012.60 22648185

[12] Tan J, Peng Q, Li J, Guan Y, Zhang L, Jiao Y, et al. Characteristics of dental morphology in the Xinjiang Uyghurs and correlation with the EDARV370A variant. Sci China Life Sci. 2014;57(5):510–8. doi: 10.1007/s11427-014-4654-x 24752358

[13] Kimura R, Yamaguchi T, Takeda M, Kondo O, Toma T, Haneji K, et al. A common variation in EDAR is a genetic determinant of shovel-shaped incisors. Am J Hum Genet. 2009;85(4):528–35. doi: 10.1016/j.ajhg.2009.09.006 19804850 PMC2756549

[14] Mou C, Thomason HA, Willan PM, Clowes C, Harris WE, Drew CF, et al. Enhanced ectodysplasin-A receptor (EDAR) signaling alters multiple fiber characteristics to produce the East Asian hair form. Hum Mutat. 2008;29(12):1405–11. doi: 10.1002/humu.20795 18561327

[15] Sabeti PC, Varilly P, Fry B, Lohmueller J, Hostetter E, Cotsapas C, et al. Genome-wide detection and characterization of positive selection in human populations. Nature. 2007;449(7164):913–8. doi: 10.1038/nature06250 17943131 PMC2687721

[16] Scott GR, Turner CG. The anthropology of modern human teeth: dental morphology and its variation in recent human populations. Cambridge; New York: Cambridge University Press; 1997. xxiii, 382 p. p.

[17] Quinn GP. Improving cancer clinical research and trials with Hispanic populations: training and outreach efforts between Moffitt Cancer Center and the Ponce School of Medicine. Rev Recent Clin Trials. 2014;9(4):223–4. 25766972 PMC5245171

[18] DeSantis CE, Ma J, Goding Sauer A, Newman LA, Jemal A. Breast cancer statistics, 2017, racial disparity in mortality by state. CA Cancer J Clin. 2017;67(6):439–48. doi: 10.3322/caac.21412 28972651

[19] Yedjou CG, Tchounwou PB, Payton M, Miele L, Fonseca DD, Lowe L, et al. Assessing the Racial and Ethnic Disparities in Breast Cancer Mortality in the United States. Int J Environ Res Public Health. 2017;14(5). doi: 10.3390/ijerph14050486 28475137 PMC5451937

[20] Wu AH, Gomez SL, Vigen C, Kwan ML, Keegan TH, Lu Y, et al. The California Breast Cancer Survivorship Consortium (CBCSC): prognostic factors associated with racial/ethnic differences in breast cancer survival. Cancer Causes Control. 2013;24(10):1821–36. doi: 10.1007/s10552-013-0260-7 23864487 PMC4046898

[21] Tannenbaum SL, Koru-Sengul T, Miao F, Byrne MM. Disparities in survival after female breast cancer diagnosis: a population-based study. Cancer Causes Control. 2013;24(9):1705–15. doi: 10.1007/s10552-013-0246-5 23775026

[22] Smigal C, Jemal A, Ward E, Cokkinides V, Smith R, Howe HL, et al. Trends in breast cancer by race and ethnicity: update 2006. CA Cancer J Clin. 2006;56(3):168–83. doi: 10.3322/canjclin.56.3.168 16737949

[23] Zhu Y, Sidell MA, Arterburn D, Daley MF, Desai J, Fitzpatrick SL, et al. Racial/Ethnic Disparities in the Prevalence of Diabetes and Prediabetes by BMI: Patient Outcomes Research To Advance Learning (PORTAL) Multisite Cohort of Adults in the U.S. Diabetes Care. 2019;42(12):2211–9. doi: 10.2337/dc19-0532 31537541 PMC6868463

[24] Shaibi GQ, Coletta DK, Vital V, Mandarino LJ. The design and conduct of a community-based registry and biorepository: a focus on cardiometabolic health in Latinos. Clin Transl Sci. 2013;6(6):429–34. doi: 10.1111/cts.12114 24119012 PMC4225082

[25] Shaibi G, Singh D, De Filippis E, Hernandez V, Rosenfeld B, Otu E, et al. The Sangre Por Salud Biobank: Facilitating Genetic Research in an Underrepresented Latino Community. Public Health Genomics. 2016;19(4):229–38. doi: 10.1159/000447347 27376364 PMC5892419

[26] Patel BK, Ridgeway JL, Ghosh K, Rhodes DJ, Borah B, Jenkins S, et al. Behavioral and psychological impact of returning breast density results to Latinas: study protocol for a randomized clinical trial. Trials. 2019;20(1):744. doi: 10.1186/s13063-019-3939-6 31852492 PMC6921571

[27] DeMenna J, Puppala S, Chittoor G, Schneider J, Kim JY, Shaibi GQ, et al. Association of common genetic variants with diabetes and metabolic syndrome related traits in the Arizona Insulin Resistance registry: a focus on Mexican American families in the Southwest. Hum Hered. 2014;78(1):47–58. doi: 10.1159/000363411 25060389 PMC4910511

[28] Yan M, Wang LC, Hymowitz SG, Schilbach S, Lee J, Goddard A, et al. Two-amino acid molecular switch in an epithelial morphogen that regulates binding to two distinct receptors. Science. 2000;290(5491):523–7. doi: 10.1126/science.290.5491.523 11039935

[29] Neel JV. Diabetes mellitus: a "thrifty" genotype rendered detrimental by "progress"? Am J Hum Genet. 1962;14:353–62. 13937884 PMC1932342

[30] Wittke-Thompson JK, Pluzhnikov A, Cox NJ. Rational inferences about departures from Hardy-Weinberg equilibrium. Am J Hum Genet. 2005;76(6):967–86. doi: 10.1086/430507 15834813 PMC1196455

[31] Turner S, Armstrong LL, Bradford Y, Carlson CS, Crawford DC, Crenshaw AT, et al. Quality control procedures for genome-wide association studies. Curr Protoc Hum Genet. 2011;Chapter 1:Unit1 19. doi: 10.1002/0471142905.hg0119s68 21234875 PMC3066182

[32] Masarwah A, Auvinen P, Sudah M, Rautiainen S, Sutela A, Pelkonen O, et al. Very low mammographic breast density predicts poorer outcome in patients with invasive breast cancer. Eur Radiol. 2015;25(7):1875–82. doi: 10.1007/s00330-015-3626-2 25735512

[33] Sprague BL, Gangnon RE, Burt V, Trentham-Dietz A, Hampton JM, Wellman RD, et al. Prevalence of mammographically dense breasts in the United States. J Natl Cancer Inst. 2014;106(10). doi: 10.1093/jnci/dju255 25217577 PMC4200066

[34] Lira FS, Rosa JC, Cunha CA, Ribeiro EB, do Nascimento CO, Oyama LM, et al. Supplementing alpha-tocopherol (vitamin E) and vitamin D3 in high fat diet decrease IL-6 production in murine epididymal adipose tissue and 3T3-L1 adipocytes following LPS stimulation. Lipids Health Dis. 2011;10:37. doi: 10.1186/1476-511X-10-37 21352586 PMC3050762

[35] Ding C, Gao D, Wilding J, Trayhurn P, Bing C. Vitamin D signalling in adipose tissue. Br J Nutr. 2012;108(11):1915–23. doi: 10.1017/S0007114512003285 23046765

[36] Rosenstreich SJ, Rich C, Volwiler W. Deposition in and release of vitamin D3 from body fat: evidence for a storage site in the rat. J Clin Invest. 1971;50(3):679–87. doi: 10.1172/JCI106538 4322721 PMC291976

[37] Mawer EB, Backhouse J, Holman CA, Lumb GA, Stanbury SW. The distribution and storage of vitamin D and its metabolites in human tissues. Clin Sci. 1972;43(3):413–31. doi: 10.1042/cs0430413 4342673

[38] Martinaityte I, Kamycheva E, Didriksen A, Jakobsen J, Jorde R. Vitamin D Stored in Fat Tissue During a 5-Year Intervention Affects Serum 25-Hydroxyvitamin D Levels the Following Year. J Clin Endocrinol Metab. 2017;102(10):3731–8. doi: 10.1210/jc.2017-01187 28973683

[39] Didriksen A, Burild A, Jakobsen J, Fuskevag OM, Jorde R. Vitamin D3 increases in abdominal subcutaneous fat tissue after supplementation with vitamin D3. Eur J Endocrinol. 2015;172(3):235–41. doi: 10.1530/EJE-14-0870 25661743

[40] Ching S, Kashinkunti S, Niehaus MD, Zinser GM. Mammary adipocytes bioactivate 25-hydroxyvitamin D(3) and signal via vitamin D(3) receptor, modulating mammary epithelial cell growth. J Cell Biochem. 2011;112(11):3393–405. doi: 10.1002/jcb.23273 21769914 PMC3196822

[41] Johnson AL, Zinser GM, Waltz SE. Loss of vitamin D receptor signaling from the mammary epithelium or adipose tissue alters pubertal glandular development. Am J Physiol Endocrinol Metab. 2014;307(8):E674–85. doi: 10.1152/ajpendo.00200.2014 25139050 PMC4200307

[42] Yang J, Zhou W, Zhu J, Wu Y, Xu L, Wang Y, et al. Circulating ectodysplasin A is a potential biomarker for nonalcoholic fatty liver disease. Clin Chim Acta. 2019;499:134–41. doi: 10.1016/j.cca.2019.09.009 31526774

[43] Garrison NA, Cho MK. Awareness and Acceptable Practices: IRB and Researcher Reflections on the Havasupai Lawsuit. AJOB Prim Res. 2013;4(4):55–63. doi: 10.1080/21507716.2013.770104 24089655 PMC3786163

