# Supplementary material for: Association of EDARV370A with breast density and metabolic syndrome in Latinos
Source: PLoS One. 2021 Oct 7;16(10):e0258212. doi: 10.1371/journal.pone.0258212 (PMC8496850; doi:10.1371/journal.pone.0258212)
Supplement: S1 Table — Values are mean ± SEM. (PDF) [file pone.0258212.s001.pdf]

S1 Table. AIR registry characteristic data organized by EDARV370A genotype and body mass index (BMI)

	Lean (BMI < 25 kg/m2)			Overweight (BMI 25-29.9 kg/m2)			Obese (≥ 30 kg/m2)		
	**AA**	**AG**	**GG**	**AA**	**AG**	**GG**	**AA**	**AG**	**GG**
Gender (Female / Male)	20F/9M	32F/12M	10F/9M	32F/21M	48F/37M	26F/23M	40F/17M	88F/36M	25F/12M
Age, years	28.7 ± 1.9	31.6 ± 1.7	31.1 ± 2.5	37.1 ± 1.5	37.4 ± 1.1	40 ± 1.5	38.8 ± 1.6	37.5 ± 1	34.8 ± 1.6
Body Mass Index, kg/m2	22.1 ± 0.4	22.6 ± 0.3	22.5 ± 0.6	27.5 ± 0.2	27.7 ± 0.1	27.5 ± 0.2	34.6 ± 0.6	34.9 ± 0.4	34.9 ± 0.6
Fat Mass, %	11.8 ± 0.7	13.9 ± 0.6	13 ± 1.1	20.6 ± 0.8	20.1 ± 0.5	19 ± 0.7	31.5 ± 1.1	33 ± 0.9	32.6 ± 1.1
Waist circumference, cm	78.9 ± 1.1	81.3 ± 0.9	84 ± 2.0	93.4 ± 0.8	94.9 ± 0.8	94.6 ± 0.9	108.2 ± 1.5	108.8 ± 1	107.3 ± 1.4
Hip circumference, cm	95.1 ± 0.7	96.7 ± 1.1	95.7 ± 1.8	104.1 ± 0.7	104.3 ± 0.5	103.4 ± 0.7	117.1 ± 1.3	117.3 ± 1	116.8 ± 1.3
Cholesterol, mg/dl	153.1 ± 4.8	166.8 ± 6.1	157.3 ± 8.0	178.5 ± 4.5	174.5 ± 3.6	185.9 ± 5.8	173.5 ± 4.1	178.3 ± 3	177.1 ± 6.2
Triglycerides, mg/dl	81.1 ± 4.6	99.4 ± 7.3	98.7 ± 11.7	121.8 ± 8.7	128 ± 7.1	174.7 ± 15.5	138 ± 9.4	155.9 ± 7.3	160.6 ± 14
High-density lipoprotein, mg/dl	49.7 ± 2.0	50.3 ± 1.9	43.2 ± 1.9	46.3 ± 1.5	44.5 ± 1.3	42.6 ± 1.6	42 ± 1.6	41.5 ± 0.8	42 ± 1.6
Low-density lipoprotein, mg/dl	90 ± 4.3	97.1 ± 4.8	97.7 ± 6.6	111.6 ± 3.8	106.5 ± 3.0	114.9 ± 4.8	108.3 ± 3.5	110.9 ± 2.6	107.3 ± 4.5
Very low-density lipoprotein, mg/dl	13.6 ± 0.7	16.7 ± 1.2	16.5 ± 2.0	20.3 ± 1.5	21.4 ± 1.2	25 ± 1.7	23.1 ± 1.6	24.8 ± 1	25.1 ± 1.6
Systolic blood pressure, mm Hg	114 ± 2.7	114.5 ± 1.9	111.3 ± 2.4	117.4 ± 1.8	117.1 ± 1.3	118.9 ± 1.8	125.9 ± 2.1	124.4 ± 1.5	121.9 ± 2.4
Diastolic blood pressure, mm Hg	72.2 ± 2.0	72.7 ± 1.3	70.3 ± 2.0	75.9 ± 1.3	74.4 ± 0.9	75.9 ± 1.1	80.5 ± 1.4	78.7 ± 0.9	78.6 ± 1.5
Alanine aminotransferase, IU/L	20.0 ± 3.1	17.3 ± 1.2	27.8 ± 4.4	23.3 ± 2.2	25 ± 1.4	30 ± 3.3	26.4 ± 1.7	30.1 ± 1.8	32 ± 2.9
Aspartate aminotransferase, IU/L	22.6 ± 2.0	21.4 ± 1.1	25.8 ± 2.5	22.2 ± 1.4	22.4 ± 0.9	24.4 ± 1.7	24.5 ± 1.5	25.6 ± 1.1	27.2 ± 1.7
Adiponectin, µg/ml	7.3 ± 0.6	8.1 ± 0.4	7.7 ± 0.9	6.3 ± 0.3	6.2 ± 0.3	5.9 ± 0.3	6.2 ± 0.3	6 ± 0.2	5.7 ± 0.3
Hemoglobin A1c, %	5.36 ± 0.05	5.44 ± 0.04	5.55 ± 0.06	5.54 ± 0.05	5.58 ± 0.03	5.58 ± 0.05	5.68 ± 0.05	5.67 ± 0.03	5.64 ± 0.07
Fasting plasma insulin, µIU/ml	5.1 ± 0.6	5.7 ± 0.6	5.2 ± 0.5	6.8 ± 0.6	7.9 ± 0.5	8.7 ± 1.0	13.5 ± 1.8	12.3 ± 0.8	10.2 ± 1.1
Fasting plasma glucose, mg/dl	90.9 ± 2.5	89 ± 1.2	93.4 ± 2.7	92.1 ± 1.4	92.2 ± 0.9	96.2 ± 2.3	96.2 ± 1.3	97.2 ± 1.2	97.7 ± 1.9
2hOGTT, mg/dl	119.8 ± 7.1	114 ± 5.3	140.1 ± 13.3	123.8 ± 5.4	124.4 ± 4.0	147.1 ± 9.3	135.4 ± 7.6	153 ± 4.9	154 ± 8.4
Diabetes status, %	7.1 ± 5.0	4.9 ± 3.0	10.5 ± 7.0	9.4 ± 4	7.1 ± 3	29.8 ± 7	16.7 ± 5	20.5 ± 4	18.9 ± 7
Prediabetes status, %	26.9 ± 9.0	15.4 ± 6.0	23.5 ± 11	25 ± 6	34.2 ± 5	36.4 ± 9	35.6 ± 7	50.5 ± 5	56.7 ± 9

Values are mean ± SEM. Characteristic data is organized by genotype and body mass index category. BMI categorization was not available for 5 of the participants.
