# Supplementary material for: Association of EDARV370A with breast density and metabolic syndrome in Latinos
Source: PLoS One. 2021 Oct 7;16(10):e0258212. doi: 10.1371/journal.pone.0258212 (PMC8496850; doi:10.1371/journal.pone.0258212)
Supplement: S2 Table — Values are mean ± SEM. (PDF) [file pone.0258212.s002.pdf]

S2 Table. SPS biobank characteristic data organized by EDARV370A genotype and body mass index (BMI)

	**Lean (BMI < 25 kg/m2)**			**Overweight (BMI 25-29.9 kg/m2)**			**Obese (≥ 30 kg/m2)**		
	**AA**	**AG**	**GG**	**AA**	**AG**	**GG**	**AA**	**AG**	**GG**
Gender (Female / Male)	22F/11M	51F/14M	19F/14M	55F/41M	124F/42M	50F/40M	121F/48M	154F/59M	91F/34M
Age, years	43 ± 2.6	43.1 ± 1.6	41.6 ± 2.3	44.3 ± 1.3	45.8 ± 0.8	45.1 ± 1.4	47.3 ± 0.9	47.5 ± 0.8	45.4 ± 1.0
Body Mass Index, kg/m2	22.1 ± 0.3	22.4 ± 0.2	22.2 ± 0.4	27.2 ± 0.2	27.2 ± 0.1	27.1 ± 0.1	34.9 ± 0.4	35.2 ± 0.4	34.6 ± 0.4
Waist circumference, cm	82.4 ± 1.3	81.7 ± 0.8	84.4 ± 1.5	93.6 ± 0.8	93.5 ± 0.5	94 ± 0.7	110.5 ± 1.1	110.6 ± 0.9	109.6 ± 1.3
Cholesterol, mg/dl	183.9 ± 7.3	188.6 ± 4.9	181.8 ± 5.2	190.6 ± 3.9	189.1 ± 2.7	189.9 ± 4.2	184.4 ± 2.7	184.1 ± 2.6	184.3 ± 2.9
Triglycerides, mg/dl	107.6 ± 10.9	97.8 ± 6.4	111.9 ± 11.2	132.8 ± 8.6	152.2 ± 8.0	144.9 ± 8.4	160.8 ± 9.5	155 ± 5.9	147.6 ± 6.9
High-density lipoprotein, mg/dl	55.4 ± 3.1	62.1 ± 2.3	60.1 ± 2.5	51.1 ± 1.4	51 ± 1.4	48.4 ± 1.2	46.6 ± 0.9	47.3 ± 0.9	47.2 ± 1.1
Low-density lipoprotein, mg/dl	107 ± 6.0	107 ± 4.4	99.2 ± 4.5	113.6 ± 3.1	109.4 ± 2.2	112.6 ± 3.6	107.2 ± 2.4	105.9 ± 2.2	107.5 ± 2.5
Hemoglobin A1c, %	5.74 ± 0.11	5.83 ± 0.15	5.99 ± 0.29	5.95 ± 0.14	6.1 ± 0.11	6.05 ± 0.14	6.05 ± 0.08	6.31 ± 0.09	6.34 ± 0.13
Fasting plasma insulin, μIU/ml	3.8 ± 0.3	3.8 ± 0.3	4.5 ± 0.9	6.5 ± 0.6	7.6 ± 1.0	7.6 ± 1.2	12.7 ± 1.4	16.6 ± 2.4	11.5 ± 0.8
Fasting plasma glucose, mg/dl	87.8 ± 1.3	86.9 ± 1.1	90 ± 2.2	91.3 ± 0.9	95.3 ± 2.3	94.1 ± 1.5	97.5 ± 1.5	96.5 ± 1.3	101.1 ± 3.5
2hOGTT, mg/dl	105.9 ± 5.8	97.3 ± 3.9	99.1 ± 5.6	113.5 ± 4.4	120.8 ± 5.2	111.9 ± 3.8	128.9 ± 4.1	134.5 ± 4.4	134.2 ± 7.5

Values are mean ± SEM. Characteristic data is organized by genotype and body mass index category.
For the SPS biobank, we were able to call 993 genotypes out of the 997 DNA samples.
BMI categorization was not available for 3 of the participants.
