# Supplementary material for: Association of EDARV370A with breast density and metabolic syndrome in Latinos
Source: PLoS One. 2021 Oct 7;16(10):e0258212. doi: 10.1371/journal.pone.0258212 (PMC8496850; doi:10.1371/journal.pone.0258212)
Supplement: S3 Table — (PDF) [file pone.0258212.s003.pdf]

S3 Table. Gender and genotype class specific mean values for the glycemic traits in the combined AIR registry + SPS biobank

**Phenotype**	**Gender**	**EDARwt / EDARwt (AA)**	**EDARwt / EDARV370A (AG)**	**EDAR370A / EDARV370A (GG)**	**P Value***	**P Value****
FPG	All	94.2 ± 0.7	94.4 ± 0.7	96.9 ± 1.4	0.062	**0.036**
FPG	Females only	92.8 ± 0.7	93.1 ± 0.7	97.5 ± 2.1	**0.0095**	**0.0052**
FPG	Males only	96.7 ± 1.6	97.7 ± 1.9	96.0 ± 1.0	0.799	0.897
HbA1c	All	5.86 ± 0.05	5.98 ± 0.04	6.06 ± 0.07	**0.018**	**0.010**
HbA1c	Females only	5.85 ± 0.05	5.92 ± 0.04	6.07 ± 0.09	**0.0134**	**0.0053**
HbA1c	Males only	5.88 ± 0.11	6.12 ± 0.12	6.04 ± 0.12	0.358	0.341

The p values were generated using the simple linear regression model in R. *Genotype only was included in the linear regression model. **Genotype, age and BMI were included in the linear regression model. Significant p values are bolded.
