# Supplementary material for: Association of EDARV370A with breast density and metabolic syndrome in Latinos
Source: PLoS One. 2021 Oct 7;16(10):e0258212. doi: 10.1371/journal.pone.0258212 (PMC8496850; doi:10.1371/journal.pone.0258212)
Supplement: S4 Table — Values are mean ± SEM. (PDF) [file pone.0258212.s004.pdf]

**S4 Table. Genotype class specific mean values for EDA-A2 levels**

| Phenotype | EDARwt / EDARwt (AA) | EDARwt / EDARV370A (AG) | EDAR370A / EDARV370A (GG) | P Value* | P Value** |
|---|---|---|---|---|---|
| EDA-A2 (pg/ml) | 80.5 ± 28.1 | 137.0 ± 36.0 | 56.3 ± 25.0 | 0.587 | 0.689 |

The p values were generated using the simple linear regression model in R. *Genotype only was included in the linear regression model. **Genotype, age, sex and BMI were included in the linear regression model.
